# The Scientific Artizan

**Published:** 1859-02

**Authors:** 


### THE SCIENTIFIC ARTIZAN.

A Journal devoted to Science, Art, Discovery, and Invention, published weekly,—price, $2 a year; $1 in advance; and $1 at the end of six months.

It is the representative of the American Patent Company, recently established in this city, and composed of respectable, wealthy, influential and scientific men. The paper contains, each week, a full report of all patents issued during preceding week, and is filled with interesting scientific information on almost every subject, and is therefore desirable and valuable, alike to the literary, scientific and professional, or the inventor, mechanic, and agriculturist,—each will be well repaid for its regular perusal; and those who have improvements, and design patenting them, will do well to consult the American Patent Company, before taking steps in the matter; they will thus be able to learn—1st, whether a patent can be obtained, 2d, whether it will be valid and can be sustained, and 3d, whether its merits will justify the labor and expense.

The following we copy from the first number, in which will be found something of the objects and aims of the Company:

"The American Patent Company proposes:—

1. To *procure patents* with the greatest possible dispatch, and the least necessary expense.

2. To *negotiate the sale* of such patents, as upon due testing prove to be worthy of public patronage.

3. To *test*, by actual experiment, the practicability and utility of all patents coming into its hands.

4. To *investigate Patent Claims*, so as to determine whether a discovery or application is patentable or not.

5. To *execute for inventors*, mechanical and other drawings, in the highest style of art, and with absolute correctness.

6. To *introduce new inventions* into general use, by presenting their claims through the columns of this paper.

7. To furnish the most finished engraving for the use of patentees at the lowest price.

8. To publish a weekly Scientific Journal, embodying all information pertaining to the patent business.

To make the objects of the Company perfectly clear, we beg to discuss briefly each of these separate departments, and to examine its claims to public confidence.

The Company is composed of gentlemen of well-known business capacity, and high reputation for integrity, possessing capital and influence. An examination of their names will show that these are not the men to engage in an illegitimate speculation. They have organized themselves under the law as a body corporate and politic, with all the rights and privileges usually accorded to such corporations. The capital stock is ONE HUNDRED THOUSAND DOLLARS, which will be increased if it be found to be necessary in the prosecution of its business.

